# Clinicopathological and genetic features of Chinese hereditary nonpolyposis colorectal cancer (HNPCC)

**DOI:** 10.1007/s12032-014-0223-1

**Published:** 2014-09-13

**Authors:** Fangqi Liu, Li Yang, Xiaoyan Zhou, Weiqi Sheng, Sanjun Cai, Lei Liu, Peng Nan, Ye Xu

**Affiliations:** 1 Department of Colorectal Surgery, Fudan University Shanghai Cancer Center, Shanghai, 200032 China; 2 Department of Oncology, Shanghai Medical College, Fudan University, Shanghai, 200032 China; 3 Department of Pathology, Fudan University Shanghai Cancer Center, Shanghai, 200032 China; 4 Ministry of Education Key Laboratory for Biodiversity Science and Ecological Engineering, School of Life Sciences, Fudan University, Shanghai, 200433 China; 5 Shanghai Medical College of Fudan University, Shanghai, 200032 China

**Keywords:** HNPCC, Clinicopathological features, MLH1/MSH2 mutations, Clinical criteria

## Abstract

The aim of this study was to investigate the clinical value of different criteria and to understand the relationship between genotype and phenotype in Chinese hereditary nonpolyposis colorectal cancer (HNPCC). A total of 116 unrelated probands of suspected HNPCC families from the Fudan Colorectal Registry were studied. A total of 32, 28, and 56 families fulfilled the Amsterdam criteria, the Fudan criteria and the revised Bethesda guideline, respectively. Direct DNA sequencing of all exons of hMSH2 and hMLH1 genes were performed on all 116 samples. Mutations and clinicopathological features were compared between the groups. Thirty-two pathological germline mutations were identified. Out of 32 mutations, 16 were located at hMLH1 and 16 at hMSH2. The sensitivity of Amsterdam criteria was 50 %, specificity was 81 %, and Youden’s index was 31 %. The sensitivity of Fudan criteria was 75 %, specificity was 58 %, and Youden’s index was 33 %. Among all the 32 families with mutations, families with hMSH2 mutation had a higher ratio of synchronous and metachronous colon cancers than families with hMLH1 mutation (33 vs. 6 %, *P* = 0.04). Patients with hMSH2 mutation more frequently harbour synchronous and metachronous colon cancers. Fudan criteria had a little higher sensitivity and accuracy than Amsterdam criteria for identification of Chinese HNPCC.

## Introduction

Hereditary nonpolyposis colorectal cancer (HNPCC), also known as Lynch Syndrome, is an autosomal-dominant syndrome accounting for 3–5 % of colorectal cancer cases [1, 2]. Germline mutations resulting in HNPCC have been found in six mismatch repair (MMR) genes (hMLH1, hMSH2, hMSH3, hMSH6, hPMS1, and hPMS2) [3–6]. Most (90 %) of the mutations locate in two MMR genes, MLH1 (50 %) and MSH2 (40 %) [7]. Lynch viewed HNPCC as a syndrome characterized by an autosomal-dominant pattern of inheritance, early onset of malignancy with a predilection for the proximal colon, multiple CRCs, the absence of premonitory lesions (e.g. adenomas), and the occurrence of cancer in certain extracolonic sites, notably endometrium and ovary [2, 8].

Traditionally, screening for HNPCC has relied on examination of family history and other clinicopathological criteria, such as the Amsterdam criteria [9]. Adding the Bethesda guidelines to the diagnostic process allowed the identification of more individuals likely to have HNPCC [10, 11]. However, the Bethesda guidelines are not highly specific. Studies of families meeting the Amsterdam criteria have identified germline MSH2 and MLH1 mutations with a relatively high sensitivity (~60 %) and specificity (~70 %). In contrast, germline MSH2 and MLH1 mutations were found with a higher sensitivity (~94 %) and a lower specificity (~30 %) when families meeting the Bethesda criteria were studied [12]. Compared with these two criteria, one of the differences is extracolonic cancers types. Stomach, ovary and brain cancers were not included in the Amsterdam criteria. The extracolonic cancers of AC II only included endometrial cancer, small intestinal cancer and ureter or renal pelvis cancer, because gastric cancer was only frequently reported in Asian HNPCC families, and it is uncommon and is observed mainly in patients from older generations in Western countries. According to previous studies on HNPCC, there were many differences between the Western and the Eastern countries [13]. For example, a predominance of distal tumours was reported in Asian patients with HNPCC [14], and our previous studies showed that the primary extracolonic tumours in stomach were more frequent [15]. Gastric cancer is the second most common extracolonic malignancy in HNPCC [16], and in some Asian populations, it is even the most common extracolonic cancer [17, 18]. According to the clinical features of Asian population, Fudan University Shanghai Cancer Center suggested include gastric cancer in Amsterdam criteria II and regarded this changed standard as “Fudan criteria”, which was first mentioned in 2004 China Tumour Clinical Yearbook.

The golden standard for diagnostic of HNPCC is germline mutation in MMR genes. At present, direct sequencing is the key procedure in recognizing MMR genes defects [19]. Molecular genetic heterogeneity must be considered when assessing the Lynch syndrome cancer phenotype. It is important to appreciate that there are important differences between the different forms of Lynch syndrome. For example, Lynch syndrome-MLH1 type appears to be associated with a deficit of extracolonic cancers (such as endometrial cancers) and an excess of CRCs, when compared with Lynch syndrome-MSH2 type, which is prominently associated with extracolonic cancers. In patients with endometrial cancer, MMR gene mutations are most prevalent in the MSH2 gene (5.2–7.0 %). On the other hand, Lynch syndrome-MSH6 type is associated with later onset CRCs and a greater number of endometrial carcinomas. Lynch syndrome-PMS2 type appears to have a later age of onset of CRC, but we do not know enough about these families to provide a complete description of how they differ from the phenotypes mentioned above [20, 21]. So far, only small genotype–phenotype studies have been performed to clarify the risks for patients specific to the MMR gene affected. Prediction of clinical risks and outcomes associated with each gene has not been possible in a reliable manner.

The aim of this study is to investigate the clinical value of different criteria and to understand the relationship between genotype and phenotype in Chinese HNPCC.

## Materials and methods

### Patients

A total of 116 families with suspected HNPCC were studied from 1994 to 2008 in Shanghai Cancer Hospital of Fudan University. All were native Chinese without immigrant ancestors. Thirty-two families fulfilled Amsterdam II criteria and were classified as AC group. Among the rest 84 families, there were 28 who met the Fudan criteria were classified as FD group.

Information of the probands and their relatives, i.e. age of onset, site of the CRCs, operative notes, pathological report, synchronous or metachronous colorectal cancers, extracolonic cancers, were documented. Available information of family history was documented for each family in a fourth-generation pedigree.

### Extraction of DNA from blood

Blood samples were taken from 116 probands. Genomic DNA was isolated from peripheral blood lymphocytes according to the manufacturer’s instructions (TIANGEN BIOTECH, BEIJING). The extracted genomic DNA was stored at −20 °C for further analysis.

### Mutation of MLH1 and MSH2 genes

Exons 1–19 of MLH1 and exons 1–16 of MSH2, including the splice-site junctions, were amplified by polymerase chain reaction (TaKaKa Biotechnology, Dalian). The PCR products were purified by 1.5 % agarose gel electrophoresis, following the manufacturer’s instructions (TIANGEN BIOTECH, BEIJING). All the purified products were sequenced by Shanghai Sunny Biotechnology Co., Ltd with 3730XL of ABI. Each mutation was amplified for both the sense and antisense strand, and then, the experiment was repeated at least once. If the mutation was exactly the same through at least three repeated experiments, the mutation was confirmed. By comparing with the reference sequences, missense and frameshift mutations were identified.

### Definition of pathogenic mutations

Sequence variants that would obviously impact the function of MLH1 or MSH2 proteins, such as nonsense and frameshift mutations, were considered pathogenic. Besides, the mutations at highly conserved splice sites were also considered pathogenic. All the missense mutations were assessed for pathogenicity by searching against the InSiGHT database (www.insight-group.org), MMR Gene Unclassified Variants Database (www.mmruv.info), Mismatch Repair Genes Variants Database (www.med.mun.ca/MMRvariants), and the Human Gene Mutation Database (www.hgmd.cf.ac.uk/ac). If the missense mutations affected the promoter site, or have been reported as pathogenic mutations in other studies, they were also considered pathogenic. Those missense mutations whose pathogenicity could not be confirmed were classified as uncertain.

### Statistical analysis

Differences in the frequency distributions of categorical factors, sex, age at diagnosis, tumour location and pathology, were compared between Amsterdam I/II criteria and Fudan criteria using partitions of the chi-square test. All the statistical analysis was carried out using SPSS statistical software (version 15.0). A *P* value of < 0.05 was considered statistically significant.

## Results

### MLH1 and MSH2 mutations

Thirty-two pathological germline mutations were identified. Sixteen mutations were located at hMLH1, and 16 were located at hMSH2. There was a wide spectrum of mutation type, including 14 missense, 7 nonsense, 4 splice site, 2 frame insertion or deletion, and 5 frame shift mutations. Among the 16 mutations of hMLH1, exon12 and exon15 both occupied three mutations. Among the 16 mutations of hMLH2, exon7 had six.

### Amsterdam criteria (AC group) and Fudan criteria (FD group)

Compared the clinicopathological features between families belonging to these two groups, only two significant differences were discovered. More synchronous and metachronous multiple relative tumours (21.6 vs. 6 %, *P* = 0.001) were found in AC group, while more extracolorectal tumours (55.8 vs. 18.3 %, *P* = 0.000)were found in FD group. Other features such as earlier age of onset for all colorectal cancers and all tumours, the ratio of proximal colonic cancers, the ratio of synchronous and metachronous multiple colon cancers, the ratio of mucinous carcinoma and the ratio of stage III/IV colon cancer were similar (Table 1). Sixteen mutations were detected in 32 families of AC group. Using Fudan criteria, 24 mutations were detected in 60 families. The sensitivity of Amsterdam Criteria was 50 %, specificity was 81 %, and Youden’s index was 31 %. The sensitivity of Fudan criteria was 75 %, specificity was 58 %, and Youden’s index was 33 %.

**Table 1:** AC group versus FD group (CRC; colorectal cancer)

	Amsterdam criteria 32	Fudan criteria	*P* value
Families		28	
Related cancer patients/related cancers	139/191	112/120	
CRC cancer patients/CRC cancers	114/155	50/53	
Male/female	82/56	63/49	
Age of first CRC	36.5	46.6	0.530
Age of first related cancers	35.4	41.7	0.976
Right colon cancer	75/155 (48.4 %)	24/53 (45.3 %)	0.207
Synchronous or metachronous colorectal cancers	26/114 (22.8 %)	3/50 (6 %)	0.255
Synchronous or metachronous related cancers	30/139 (21.6 %)	7/112 (6 %)	0.001
Extracolonic cancers	35/191 (18.3 %)	67/120 (55.8 %)	0.000
Mucinous adenocarcinoma	9/27 (33.3 %)	4/26 (15.4 %)	0.868
Stage III/IV CRCs	8/27 (29.6 %)	6/26 (23.1 %)	0.550

### Relationship between genotype and phenotype

In families of AC group, we divided them into group A (with MMR mutation) and group B (without MMR mutation); there were no significant clinicopathological differences between these two groups (Table 2).

**Table 2:** Mutation versus non-mutation in Amsterdam criteria group

	Mutation	Non-mutation	*P* value
Families	16	16	
Related cancer patients/related cancers	73/107	66/84	
CRC cancer patients/CRC cancers	60/86	54/69	
Male/female	41/31	41/25	
Age of first CRC	36.9	36.1	0.738
Age of first related cancers	34.8	36.1	0.932
Right colon cancer	47/86 (54.7 %)	28/69 (40.6 %)	0.054
Synchronous or metachronous colorectal cancers	16/60 (26.7 %)	10/54 (18.5 %)	0.530
Synchronous or metachronous related cancers	20/73 (27.4 %)	10/66 (15.2 %)	0.172
Extracolonic cancers	19/107 (17.8 %)	16/66 (24.2 %)	0.574
Mucinous adenocarcinoma	6/13 (46.2 %)	3/14 (21.4 %)	0.234
Stage III/IV CRCs	4/13 (30.8 %)	4/14 (28.6 %)	0.730

Among all the 32 families with mutations, those with hMSH2 mutation had a higher ratio of synchronous and metachronous colon cancers than families with hMLH1 mutation (33 vs. 6 %, *P* = 0.04) (Table 3).

**Table 3:** MLH1 mutation versus MSH2 mutation

	MLH1	MSH2	*P* value
Families	16	16	
Related cancer patients/related cancers	66/86	61/88	
CRC cancer patients/CRC cancers	47/59	45/65	
Male/female	38/28	36/24	
Age of first CRC	43.4	37.9	0.166
Age of first related cancers	40.3	37.1	0.419
Right colon cancer	37/59 (62.7 %)	34/65 (52.3 %)	0.748
Synchronous or metachronous colorectal cancers	3/47 (6 %)	15/45 (33.3 %)	0.04
Synchronous or metachronous related cancers	14/66 (21.2 %)	18/61 (29.5 %)	0.433
Extracolonic cancers	25/86 (29.1 %)	23/88 (26.1 %)	0.860
Mucinous adenocarcinoma	3/15 (20 %)	7/13 (53.8 %)	0.127
Stage III/IV CRCs	6/15 (40 %)	4/13 (30.8 %)	0.502

## Discussion

### Clinical features and clinical diagnostic criteria

Among the 116 probands we collected, more left colon cancers than the right were found (64.7 vs. 35.3 %), especially the rectal cancers. MMR gene mutation is regarded as the gold standard of HNPCC. So, 32 probands with MLH1 or MSH2 positive mutation and their relatives need to be analyzed in detail. The results showed that within these 32 families, 124 CRC happened, 70 were left side, and 54 were right (56.5 vs. 43.5 %), similar to the data above. The reason may depend on the higher risk of rectal cancers in China, or this difference may exactly be the Asian HNPCC feature. Chew MH et al. [14] also mentioned this different in their report. A higher predominance of right-sided CRC as reported in most HNPCC [22, 23]. However, rectal cancers were far more frequent than previously reported [24]. Timm Goecke also reported rectal cancers were remarkably frequent in 281 MLH1/MSH2 mutation carriers in German [25]. This indicates the need of more attention to rectal cancers in HNPCC patients, not only in Asian countries but also in Western countries.

In these 32 probands with MLH1 or MSH2 positive mutation and their relatives, focusing on the extracolonic tumours, 48 related cancers were happened, most of which were gastric cancer and endometrial carcinoma, 12 each (25 %). And similar results were reported in Korea and Brazil [26, 27]. It was significantly different from most European and American HNPCC families whose most frequent extracolonic cancer was endometrial cancer [8, 28, 29]. But for Asian population, gastric cancer is one of the most frequently extracolonic cancer in the current HNPCC and suspected families [30]. Some researchers pointed out that poor living standard may be the reason to explain the high frequency of gastric cancer. The frequency of cancer in individual organs can vary substantially depending upon ethnic, racial, and geographic differences [31]. In our opinion, gastric cancer may play a particular role in HNPCC families. In fact, some studies from Europe and America also supported that gastric cancer was associated with HNPCC syndrome [27, 32]. According to a study by Watson and Lynch that was performed in America, the gastric cancer risk in the familial members of HNPCC was increased 4.1-fold over the general population [33]. Some studies also show the relative risk of gastric cancer in HNPCC mutation carriers compared with the general population has been reported to be higher by 4–19-fold [34] in populations of the Western world and at least by twofold in endemic areas in Asia [17]. Aarnio et al. [35] calculated the lifetime risk of gastric cancer in mutation carriers of the HNPCC gene as 19 % in the Finnish population. These studies all gastric cancer is the main related cancer in HNPCC family.

Back to our study, 32 probands fulfilled Amsterdam I/II criteria, 16 (50 %) have the MMR mutation. If using Fudan criteria, gastric cancer plused into the related cancer of Amsterdam II criteria, 60 probands were fulfilled, 24 of them (40 %) occupied MRR mutation. The sensitivity of Amsterdam Criteria was 50 %, specificity was 81 %, and Youden’s index was 31 %. The sensitivity of the changed criteria (Fudan criteria) was 75 %, specificity was 58 %, and Youden’s index was 33 %. Although the Amsterdam criteria have been extremely successful in achieving their original purpose of providing a common nomenclature for the HNPCC syndrome for research purposes, using these criteria in the clinical realm must be done with extreme caution. Their limited sensitivity for identifying families with MSH2 and MLH1 mutations make it inappropriate to use the Amsterdam criteria as the sole criteria in choosing which patients should undergo genetic testing. Sapna Syngal et al. [12] showed that the sensitivity of the Modified Amsterdam and Amsterdam II criteria were 72 % (95 % CI 58–86) and 78 % (95 % CI 64–92), respectively. Overall, the most sensitive criteria for identifying families with pathogenic mutations were the Bethesda criteria, with a sensitivity of 94 % (95 % CI 88–100); the specificity of these criteria was 25 % (95 % CI 14–36). Obviously, greater sensitivity of the Bethesda guidelines was achieved at the expense of decreased specificity. The Bethesda Guidelines were proposed to target who should undergo tumour MSI analysis. However, the use of MSI testing in clinical practice has some major practical obstacles: MSI testing as a routine commercial clinical laboratory test is not widely available, and tumour blocks are often difficult to obtain/analyse owing to logistical and technical difficulties [36]. In our country, many HNPCC patients were found in outpatient department and they had already received the operation in local hospital, so tumour tissues were hardly got because the patients were from all of the provinces. Based on these limitations of Bethesda Guidelines, Fudan criteria can still keep the standard not too board as Bethesda criteria. Another important sense is that with the same detection rate as Amsterdam I/II criteria, more HNPCC families could be detected by using Fudan criteria.

Further more, each feature of HNPCC between Amsterdam I/II criteria and Fudan criteria were compared. Include age of onset, sex, site of the colorectal cancer, synchronous or metachronous tumours rate, and mucinous carcinoma rate, there were no significant differences, except synchronous or metachronous tumours rate and except synchronous and metachronous multiple relative tumours (21.6 vs. 6 %, *P* = 0.001) and extracolorectal tumours (55.8 vs. 18.3 %, *P* = 0.000). These differences may be caused by gastric cancer patients in Fudan criteria families. In other words, patients who fulfilled Fudan criteria have similar clinicopathological features to the patients who fulfilled Amsterdam I/II criteria.

The contents of Fudan criteria were as followed: (1) There should at least three relatives with colorectal cancer or with an extracolonic cancer: cancer of endometrium, stomach, small bowel, ureter or renal pelvis. One relative should be a first-degree relative of the other two. (2) At least two successive generations should be affected. (3) At least one tumour should be diagnosed before age 50. (4) Familial adenomatous polyposis should be excluded. (5) Tumours should be verified by histopathological examination.

### Relationship between genotype and phenotype

Approximately 85 % of genetically defined HNPCC patients have germline mutations in MLH1 and MSH2 [3]. Several investigators have tried to correlate the phenotype with the affected gene [14]. Kastrinos et al. [37] reported that MLH1 carriers (*n* = 112) had a higher prevalence of colorectal cancer (79 vs. 69 %, *P* = 0.08) and younger age of diagnosis (42.2 vs. 44.8 years, *P* = 0.03) when compared to MSH2 carriers (*n* = 173). While the prevalence of endometrial cancer in women (68/167, 41 %) was similar in both groups (36 vs. 44 %), other extracolonic cancers were more frequent in MSH2 carriers compared to MLH1 carriers (24 vs. 9 %; OR 3.2; 95 % CI 1.5–6.6; *P* = 0.001) and their families (*P*<0.001). Choi et al. [38] analyzed 32 families from the Canadian familial colorectal cancer registry. Males with MLH1 mutations exhibited a significantly higher CRC risk than females (67 vs. 35 % by age 70, *P* = 0.02), while the risk was similar in MSH2 carriers (about 54 %). The relative risk was constant with age (hazard ratio; between 5.5 and 5.1 over age 30–70) in MLH1 carriers, while the harzard ratio in MSH2 carriers decreased with age (from 13.1 at age 30 to 5.4 at age 70). In our study, families with hMSH2 mutation had a higher ratio of synchronous and metachronous colon cancers than families with hMLH1 mutation (33 vs. 6 %, *P* = 0.04). So, we suggest patient with hMSH2 mutation need more frequent colonoscopy during follow-up. It has been recently reported that pathogenic PMS2 mutation is more frequently identified (4 %) than originally expected [39]. Hendriks et al. [40] reported that the cumulative risk for Lynch associated tumours was significantly lower in MSH6 carriers when compared to MLH1 or MSH2 mutation carriers (*P* = 0.002). Watson et al. [41] reported the risk of colorectal cancer in MSH6 carriers in the Dutch HNPCC database. The median age of onset of colorectal cancer in putative mutation carriers was 10 years higher for MSH6 (54 years; 95 % CI 51–56) compared with MLH1 and MSH2 carriers (44 years; 95 % CI 43–45). MSH6 families also showed a lower incidence of colorectal cancer compared with MLH1 and MSH2 families (*P*<0.001).Therefore, in the future study on HNPCC, MSH6 and PMS2 should be considered to be included.
